# Cellular Localization and Trafficking of the Human ABCG1 Transporter

**DOI:** 10.3390/biology3040781

**Published:** 2014-11-14

**Authors:** Edward B. Neufeld, Katherine O’Brien, Avram D. Walts, John A. Stonik, Steven J. Demosky, Daniela Malide, Christian A. Combs, Alan T. Remaley

**Affiliations:** 1 Lipoprotein Metabolism Section, Cardiovascular and Pulmonary Branch, National Heart, Lung and Blood Institute, National Institutes of Health, Bethesda, MD 20892, USA; E-Mails: jstonik@verizon.net (J.A.S.); sjdjr@nhlbi.nih.gov (S.J.D.); Alan.Remaley@nih.gov (A.T.R.); 2 Lipid Trafficking Core, National Heart, Lung and Blood Institute, National Institutes of Health, Bethesda, MD 20892, USA; E-Mails: obrienk@nhlbi.nih.gov (K.O.); awalts@yahoo.com (A.D.W.); 3 NHLBI Light Microscopy Core Facility, National Institutes of Health, Bethesda, MD 20892, USA; E-Mails: dmalide@mail.nih.gov (D.M.); combsc@nih.gov (C.A.C.)

**Keywords:** ABCG1, cholesterol, cholesterol efflux, vesicular trafficking, HDL, rescretion

## Abstract

We have developed a suitable heterologous cell expression system to study the localization, trafficking, and site(s) of function of the human ABCG1 transporter. Increased plasma membrane (PM) and late endosomal (LE) cholesterol generated by ABCG1 was removed by lipoproteins and liposomes, but not apoA-I. Delivery of ABCG1 to the PM and LE was required for ABCG1-mediated cellular cholesterol efflux. ABCG1 LEs frequently contacted the PM, providing a collisional mechanism for transfer of ABCG1-mobilized cholesterol, similar to ABCG1-mediated PM cholesterol efflux to lipoproteins. ABCG1-mobilized LE cholesterol also trafficked to the PM by a non-vesicular pathway. Transfer of ABCG1-mobilized cholesterol from the cytoplasmic face of LEs to the PM and concomitant removal of cholesterol from the outer leaflet of the PM bilayer by extracellular acceptors suggests that ABCG1 mobilizes cholesterol on both sides of the lipid bilayer for removal by acceptors. ABCG1 increased uptake of HDL into LEs, consistent with a potential ABCG1-mediated cholesterol efflux pathway involving HDL resecretion. Thus, ABCG1 at the PM mobilizes PM cholesterol and ABCG1 in LE/LYS generates mobile pools of cholesterol that can traffic by both vesicular and non-vesicular pathways to the PM where it can also be transferred to extracellular acceptors with a lipid surface.

## 1. Introduction

Cholesterol plays critical roles in maintaining proper membrane structure and function through its interactions with other membrane lipids and proteins. Exquisitely complex cellular and systemic homeostatic mechanisms have evolved to maintain membrane cholesterol content within levels commensurate with normal function. Membrane cholesterol pools are in constant flux both within membranes and in transport between membranes.

A growing number of membrane proteins that reside at the plasma membrane, in late endocytic compartments, or both, have been found to play critical roles in intracellular cholesterol trafficking, including ABCA1 [1,2], ABCA3 [3,4], NPC1 [5], MLN64 [4], among others. These proteins mediate the transfer of cholesterol from a donor membrane to an acceptor membrane, or to soluble, non-membrane acceptor proteins. Little, however, is currently known about the mechanism(s) by which these proteins alter the disposition of cholesterol in donor membranes to make it available for removal. The ABCA1 transporter, one of the better characterized proteins that alters the distribution of membrane cholesterol, generates modified cholesterol and phospholipid-containing membrane domains that lipid-poor apolipoprotein acceptors such as apoA-I are competent to associate with, and then later dissociate from the cell, with concomitant removal of membrane lipids, thereby forming the nascent HDL particle [6,7].

Interest in ABCG1 began with the discovery that cholesterol as well as LXR agonists are transcriptional activators of the transporter [8,9]. Klucken *et al.* [10] first provided evidence for a possible role for ABCG1 in sterol efflux. We subsequently reported that ABCG1 resides on the cell surface as well as in late endosomes that shuttle back to the cell surface, and that ABCG1 mobilizes a pool of cholesterol on the cell surface that is distinctive from pools mobilized by ABCA1 [11]. Tall, Oram, Edwards and their respective colleagues subsequently showed that ABCG1 promotes efflux of cellular lipids to mature HDL as well as LDL, cyclodextrin and, liposomes [12,13,14].

The subcellular site(s) of ABCG1 functionality, however, remains controversial. Although many early reports using ABCG1 with a variety of tags expressed in cultured cells indicated that ABCG1 resides and functions at the PM [13,15,16], more recent studies by Edwards and colleagues have proposed an exclusive function for ABCG1 in endosomes [17]. Studies of ABCG1 KO mice revealed that ABCG1 functions in Type II pneumocytes in lamellar bodies, and in alveolar macrophage late endosomes [18]. However, the concept that ABCG1 functions at a single subcellular site is contradicted by the finding that ABCG1 exclusively localizes in pancreatic β-cell secretory granules where it functions to modulate secretory granule release, and further, was shown to play no role in cellular cholesterol efflux to extracellular acceptors in these cells [19].

We presently report that the function of human ABCG1 stably expressed in a HeLa cell line is not altered by the fusion of EGFP to the C-terminus of the transporter insofar as ABCG1-GFP enhances cellular cholesterol efflux to extracellular acceptors with a lipid surface, including HDL, LDL, and liposomes. Our studies reveal that ABCG1-mediated enhancement of cellular cholesterol efflux requires delivery of ABCG1 from its site of synthesis in the ER, to the plasma membrane and late endocytic compartments, and that ABCG1 rapidly cycles between endosomes and the cell surface. We further show that ABCG1 trapped in late endocytic compartments, in the absence of ABCG1 at the cell surface, can still enhance cellular cholesterol efflux. ABCG1 expression also increased the flux of both dextran and HDL through late endosomes/lysosomes, suggesting the possibility of a potential additional ABCG1-mediated cellular cholesterol efflux pathway involving HDL resecretion.

## 2. Experimental Section

Stable ABCG1-GFP Expression—HeLa cells were grown in AMEM (Life Technologies, Inc., Waltham, MA, USA) medium, supplemented with 10% fetal bovine serum, 2 mM glutamine, 100 IU/mL of penicillin, 100 µg/mL streptomycin, and 100 µg/mL G418. A stably transfected ABCG1-GFP HeLa cell line was established as previously described for ABCA1-GFP [1]. Enhanced GFP along with a 5 amino acid glycine linker (Quantum Biologics, Vancouver, Canada) were fused in frame to the carboxyl terminus of human ABCG1, after first deleting the stop codon from the full-length ABCG1 cDNA. Briefly, HeLa AAb pTk-Hyg (Tet-off) cells (Palo Alto, CA, USA) were co-transfected with ExGen 500 (MBI, Fermentis, Pittsburgh, PA, USA) using the expression plasmids pTRE2-ABC8-GFP (pTRE2 (Palo Alto, CA, USA), encoding a chimeric ABCG1-GFP protein, and pTK-Hyg (Palo Alto, CA, USA). Hygromycin-resistant cells were screened for expression of the fusion protein by fluorescence microscopy and positive clones were further purified by limiting dilution. Control cells were co-transfected with pTRE2 and pTK-Hyg (Clonetech, Palo Alto, CA, USA) at a ratio of 1:20 and selected with 500 µg/mL of hygromycin.

Lipid Efflux Assays—HDL subfractions, LDL, and apoA-I were obtained from human serum by ultracentrifugation, as previously described [20]. Liposomes were prepared by sonication of L-α-phosphatidylcholine (Egg/Chicken: Avanti Polar Lipids) in PBS, as previously described [21]. For cellular lipid efflux studies, cells were grown in 24-well plates, and all assays were conducted using six replicates, and represent a minimum of three experiments. For cholesterol efflux, nearly confluent cells were labeled with 1 µCi/mL ^3^H-cholesterol for 24 h, washed, and then incubated for 4 h in AMEM containing 1 mg/mL of bovine serum albumin in the presence or absence of HDL, HDL_2_, or, HDL_3_ (50 µg/mL), LDL (50 µg/mL), PC liposomes (50 µg/mL), or, apoA-I (10 µg/mL). For phospholipid efflux, nearly confluent cells were labeled with 1 µCi/mL ^3^H-methyl-choline for 24 h, washed, and then incubated for 4 h with extracellular acceptors at the same concentrations used for cholesterol efflux. Cells were harvested by scraping, and cell-associated and medium lipids were extracted by the method of Folch [22]. Lipid-associated ^3^H was analyzed by scintillation counting. The percentage of cellular lipid effluxed was calculated by dividing the medium counts by the sum of the radioactive counts in the medium plus the cell fraction, after correction for efflux to 0.1% BSA medium.

Northern Blot Analysis—Northern analysis was performed using specific probes for ABCG1, ABCG4, and ABCA1, respectively. RNA was isolated from parental and ABCG1-GFP expressing HeLa cells with the Qiagen RNeasy Mini kit. Human brain and spleen RNA was purchased from Ambion Inc. Human ABCG1, ABCG4, and β-actin probes corresponding to unique portions of the respective genes were generated by RT-PCR and subsequently cloned and sequenced. The primers used were as follows: human ABCG1 (cctaccacaacccagcagat and ctccaggggaaatgtcagaa—491 bp probe), and human β-actin (gctccggcatgtgcaa and aggatcttcatgaggtagt—542 bp probe). Northern blots containing 10 µg of each RNA species were hybridized with ^32^P-labeled DNA probes and exposed to film. Message sizes were as predicted, namely: ABCG1-GFP—2.8 kB and B-actin—1.8 kB

Western Blot Analysis—Immunoblot analysis of SDS-PAGE gels was performed using anti-peptide ABCG1 antibodies (Gene Tex or Santa Cruz) and anti-GFP antibodies (Roche).

Endocytic Uptake—To label late endocytic compartments, cells were incubated with 0.5 mg/mL Alexa594-dextran (10 kDa) for 24 h at 37 °C in 10% FBS/AMEM medium, chased with 10% FBS/AMEM for 4 h, washed with PBS, fixed with 3% paraformaldehyde, and imaged. Cells pre-incubated with or without 60 mM sucrose for 24 h, were incubated with 50 µg/mL Alexa568-HDL for 4 h at 37 °C, washed with PBS, fixed and imaged.

Pharmacological Interventions—Various pharmacological agents were added to AMEM medium (Life Technologies, Inc.) containing 0.1% BSA for efflux studies. Cellular sphingomyelin was digested by incubation of cells with 0.1 U/mL neutral sphingomyelinase (*Staphylococcus* *aureus*, Sigma, St.Louis, Mo, USA) for the times indicated. Protein synthesis was inhibited by incubation of cells with 100 µg/mL cycloheximide. Vesicular trafficking was blocked during HDL-mediated efflux using 5 µg/mL Brefeldin A, 10 µM monensin, or, U18666A at the indicated concentrations. Sucrosome formation was induced by incubation of cells grown in AMEM (Life Technologies, Inc.) medium, supplemented with 10% fetal bovine serum, 2 mM glutamine, 100 IU/mL of penicillin, 100 µg/mL streptomycin, and 100 µg/mL G418 containing 60 mM sucrose in for 24 h.

Confocal Microscopy—Laser scanning confocal microscopy to monitor GFP, filipin and Alexa 568 and Alexa 594 fluorescence, was performed using a Zeiss 510 LSCM. Time-lapse fluorescence microscopy and 3D imaging were performed as previously described [1]. Consecutive acquisition of 3D-image stacks was used to generate time-lapse 3D images. Time-lapse movie were made using Imaris software.

Time-lapse Confocal Fluorescence Microscopy*—*Time-lapse images were taken with a Zeiss Axiovert 35 microscope equipped with a charged coupled device camera (TEA/CCD-1317K/1, Princeton Instruments, Trenton, NJ, USA). For live cell imaging, cells were prepared on 40-mm coverslips, and temperature was maintained at 37 °C in Focht Chamber System 2 with an Objective Heater System (Bioptechs, Butler, PA, USA). A total of 40 GFP images were acquired at the rate of 1/s (0.3-s exposure). Capture, animation, and export to QuickTime movie were performed using the IPLab software system (Scanalytics, Fairfax, VA, USA). Structures in movies were pseudocolored using Adobe AfterEffects 4.1 and Adobe Photoshop 5.0 software.

## 3. Results and Discussion

### 3.1. Stable Expression of GFP-Tagged Human ABCG1

**Figure 1:**
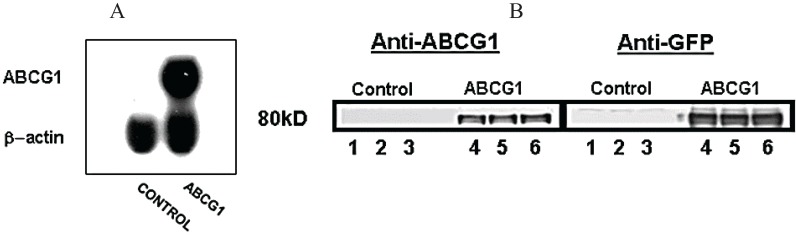
Stable expression of ABCG1-GFP in a HeLa cell line. (**A**) Northern blot analysis of control and ABCG1-GFP stably transfected HeLa cells. ABCG1-GFP is robustly expressed in the stably transfected HeLa cell line in the absence of endogenous ABCG1 expression; (**B**) Western blot analyses of control and ABCG1-GFP stably transfected HeLa cells. An 80 kD protein is recognized by antibodies against both GFP and the N-terminus of ABCG1 in stably transfected HeLa cells but not control HeLa cells, confirming ABCG1-GFP expression in the absence of endogenous ABCG1 expression.

Northern analyses revealed that the stably transfected ABCG1-GFP HeLa cell line expressed an mRNA of the expected 2.8 kB length that is recognized by an ABCG1-specific probe (Figure 1A). In contrast, ABCG1 protein was not expressed by the control parental HeLa cell line (Figure 1B), nor was ABCA1 [1] expressed in either the control or stably expressing ABCG1-GFP cell lines. The stably transfected ABCG1-GFP HeLa cell line robustly expressed a fusion protein of approximately 80 kDa that was recognized by antibodies to both N-terminal ABCG1 and GFP (Figure 1B). Thus, ABCG1-GFP is expressed in the stably expressing HeLa cell line in the absence of any detectable amounts of endogenous ABCG1 expression.

### 3.2. ABCG1 Resides on the Cell Surface and Late Endosomes

**Figure 2:**
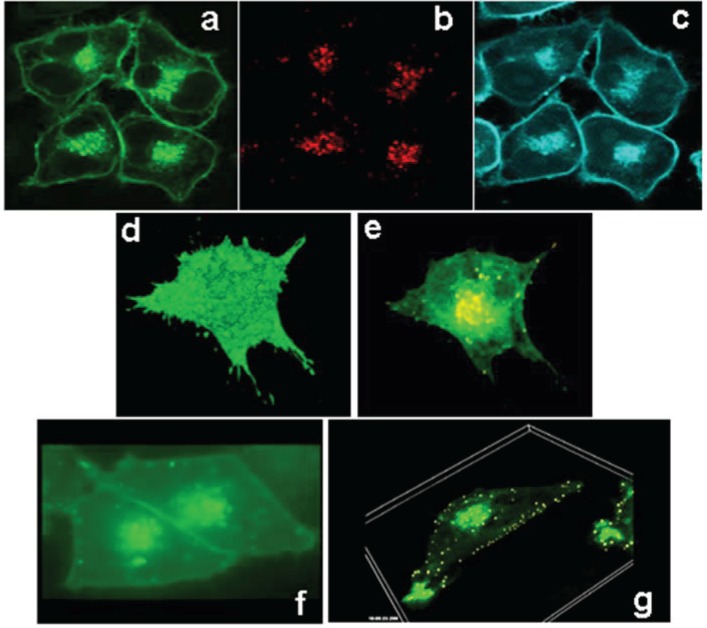
ABCG1-GFP traffics between the cell surface and late endosomes. ABCG1-GFP resides on the cell surface and intracellular vesicles (**a**; *green*), vitally labeled with endocytosed Alexa594 dextran (**b**; *red*), used as a marker for late endosomes/lysosomes. As seen in (**c**), the ABCG1 late endocytic vesicles are enriched with cholesterol as revealed by cholesterol-specific cytochemical filipin staining (*blue*). 3D reconstruction of the entire volume of an ABCG1-expressing cell shows ABCG1 in the cell surface (**d**; single frame from Movie 1). Rendering of the data set in (**d**) to reveal intracellular ABCG1 (**e**) demonstrates the colocalization of ABCG1 (*green*) in late endosomes, labeled as in (**b**) with fluorescent dextran (*red*), as *yellow* punctate structures in this merged image. Note the ABCG1 late endosomes/lysosomes localize in abundance in the perinuclear region as well as in peripheral locations close to the cell surface (**e**; single frame from Movie 2); (**f**) Time lapse confocal microscopy reveals trafficking of ABCG1-late endosomes between the perinuclear region and cell surface as well as contact of ABCG1-late endosomes with each other and the PM (Single frame from Movie 3); (**g**) 3D-Time lapse confocal microscopy shows extensive interaction of ABCG1 late endosomes with the cell surface (Single frame from Movie 4).

ABCG1-GFP stably expressed in HeLa cells resides on the cell surface (Figure 2a). The topological distribution of ABCG1 on the cell surface revealed by 3-D reconstruction of the entire volume of an ABCG1-GFP cell is shown in Figure 2d (and, Supplemental Data Movie 1). Endocytosed fluorescent dextran (Figure 2b,e, and, Supplemental Data Movie 2), used as a vital marker for late endocytic compartments [23,24], colocalized with a subset of intracellular vesicles containing ABCG1-GFP. The 3-D distribution of ABCG1 in perinuclear late endosomes, as well as peripherally located late endosomes, is shown in Figure 2e–g (and Supplemental Data Movie 2, Supplemental Data Movie 3 and Supplemental Data Movie 4). Time-lapse confocal microscopy demonstrates the localization of ABCG1 on the cell surface and the trafficking of ABCG1 in vesicles that shuttle between ABCG1-containing perinuclear late endosomes and the cell surface (Figure 2f and Supplemental Data Movie 3). 3-D time-lapse confocal microscopy revealed an extensive network of ABCG1-containing subsurface vesicles that appear to shuttle between ABCG1-containing perinuclear endosomes and the cell surface (Figure 2g and Supplemental Data Movie 4). Confocal imaging of living ABCG1-GFP cells revealed that ABCG1 endosomes make frequent contact with the PM (Movies 3, 4, 5).

### 3.3. ABCG1-GFP Expression Increases Cellular Cholesterol Efflux to Extracellular Acceptors with a Lipid Surface

**Figure 3:**
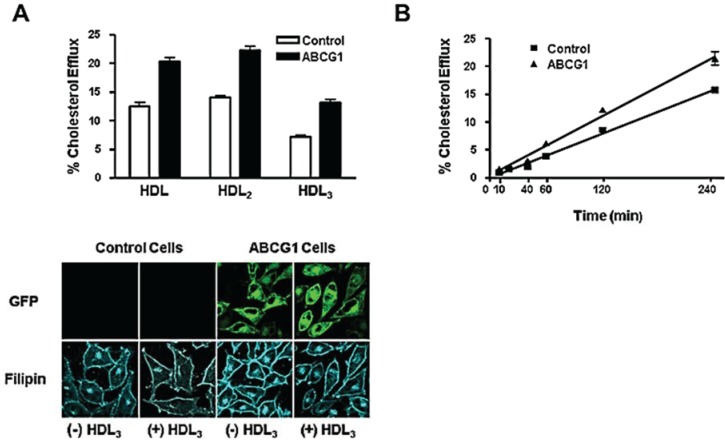
ABCG1 expression enhances high density lipoprotein (HDL)-mediated cellular cholesterol efflux. **(A)** Control and stably expressing ABCG1-GFP cells were labeled with ^3^H-cholesterol and efflux to 50 µg/mL HDL, HDL_2_, or HDL_3_ was assessed as described in “Experimental Section”. Cellular cholesterol efflux mediated by HDL, HDL_2_, and HDL_3_ was significantly increased in ABCG1-expressing cells compared to control cells (*p* < 0.0001, for HDL, HDL_2_, and HDL_3;_ unpaired two-tail *t*-tests). ABCG1 expression enhances HDL-mediated reduction in cellular cholesterol monitored by microscopy. Control and ABCG1-GFP cells were incubated in the absence ((−)HDL_3_) or presence ((+)HDL_3_) of 50 µg/mL HDL_3_ for 4 h, fixed, washed and stained with filipin as described in “Experimental Section”. Note the filipin staining is increased in ABCG1-GFP cells compared to controls and that HDL_3_ decreases filipin staining to a greater extent in ABCG1-expressing cells than in control cells; **(B)** Kinetics of ABCG1-mediated cellular cholesterol to HDL. Control and ABCG1-GFP stably expressing HeLa cells were labeled with ^3^H-cholesterol and efflux to 50 µg/mL HDL was assessed at the indicated times. All values represent mean ± S.D. Data shown is representative of at least three replicate experiments.

Stable expression of ABCG1-GFP increased cellular cholesterol efflux to HDL (150% of controls), HDL_2_ (150% of controls), and, HDL_3_ (200% of controls; Figure 3A), consistent with previous reports showing that transient overexpression of non-tagged ABCG1 enhanced cholesterol efflux to HDL lipoproteins [12,13,14]. Cytochemical analyses revealed that compared to control cells, ABCG1 expression increased cellular cholesterol on the cell surface and in late endosomes (Figure 3A; (−) HDL_3_). HDL_3_ reduced cholesterol at the cell surface and endosomes of ABCG1-GFP expressing cells, and the relative reduction was greater than that observed at these sites in control cells (Figure 3A; (+) HDL_3_). Thus, ABCG1 at the cell surface and late endosomes increases cholesterol at these sites and these ABCG1-mobilized pools of cholesterol are available for removal by HDL. Kinetic analysis (Figure 3B) revealed that cellular cholesterol efflux to HDL was linear (*r*^2^ = 0.99 for both control and ABCG1-GFP cells) and that ABCG1 increased cellular cholesterol efflux to HDL (slope = 0.066% ± 0.003% and 0.089% ± 0.004%.Total Cellular Cholesterol Efflux/min (mean ± S.D.), for control and ABCG1-GFP cells, respectively). Our findings that ABCG1 expression increases both the size of the cholesterol pool available for efflux (Figure 3A) and the rate constant for efflux (Figure 3B), is consistent with the findings of Sankaranarayanan *et al.* [25].

**Figure 4:**
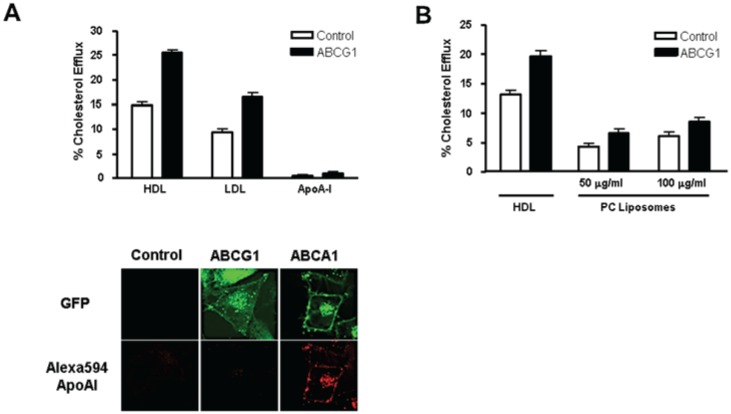
ABCG1 expression enhances cellular cholesterol efflux to extracellular acceptors with a lipid surface. (**A**) Control and ABCG1-GFP cells were labeled with ^3^H-cholesterol and efflux to 50 µg/mL HDL, LDL, or, 10 µg/mL apoA-I was assessed as described in “Experimental Section”. Note the markedly increased cellular cholesterol efflux to HDL and LDL, but not to apoA-I. Cellular cholesterol efflux mediated by HDL, LDL, and apoA-I was significantly increased in ABCG1-expressing cells compared to control cells (*p* < 0.0001, *p* < 0.0001, and *p* < 0.005, for HDL, LDL, and apoA-I, respectively_;_ unpaired two-tail *t*-tests). ABCG1 does not promote apoA-I binding or uptake. Control cells and cells stably expressing ABCA1-GFP and ABCG1-GFP were incubated with 5 µg/mL Alexa568-tagged apoA-I for 4 h, washed, fixed, and observed by confocal fluorescence microscopy as described in “Experimental Section”. Note that apoA-I binds to the cell surface and is endocytosed by HeLa cells stably expressing ABCA1-GFP, but not by control, or ABCG1-GFP-expressing cells; (**B**) ABCG1-GFP enhances cellular cholesterol efflux to PC liposomes. Control and ABCG1-GFP stably expressing HeLa cells were labeled with ^3^H-cholesterol and efflux to 50 µg/mL HDL, or PC liposomes was assessed as described in “Experimental Section”. Note that ABCG1 expression enhances liposome-mediated cellular cholesterol efflux. Cellular cholesterol efflux mediated by HDL and PC liposomes at both concentrations used was significantly increased in ABCG1-expressing cells compared to control cells (*p* < 0.0001 in all cases, unpaired two-tail *t*-tests). All values represent mean ± S.D. Data shown is representative of at least three replicate experiments.

ABCG1-expressing cells also increased efflux of cellular cholesterol to LDL, as previously reported [12] (Figure 4A). ABCG1 expression increased cellular cholesterol efflux to HDL and LDL to a similar extent (75% and 76%, for HDL and LDL, respectively). As shown in Figure 4A, ABCG1 cells did not support apoA-I-mediated cellular cholesterol efflux, and, confocal microscopy revealed that unlike ABCA1-GFP cells, ABCG1-GFP cells did not bind or endocytose apoA-I. Efflux of cellular cholesterol to liposomes, which lack apolipoprotein, was also enhanced to a similar extent in ABCG1 cells (Figure 4B). Taken together, these findings establish that the fusion of EGFP to the *C*-terminus of ABCG1 does not interfere with the function of the transporter as it is currently defined, and confirm that a lipid surface is the sole requirement for an extracellular acceptor of ABCG1 mobilized pools of cellular cholesterol.

**Figure 5:**
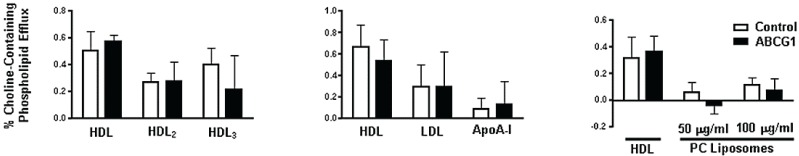
ABCG1-GFP expression does not alter cellular phospholipid efflux. Control and ABCG1-GFP stably expressing HeLa cells were labeled with ^3^H-methyl-choline and phospholipid efflux to 50 µg/mL HDL, HDL_2_, HDL_3_, LDL, or 10 µg/mL apoA-I was assessed as described in “Experimental Section”. Cellular phospholipid efflux was not significantly increased to any of the acceptors in ABCG1-expressing cells compared to control cells (*p* values ranged from *p* < 0.1 to *p* < 1.0; unpaired two-tail *t*-tests). All values represent mean ± S.D. Data shown is representative of at least three replicate experiments.

Efflux of cellular choline-containing phospholipids to HDL, HDL_2_, HDL_3_, LDL, and liposomes represented <1% of total cellular phospholipids and was not altered by ABCG1-GFP expression (Figure 5). Thus, ABCG1-mediated transfer of cell surface cholesterol to extracellular lipid-laden lipoprotein acceptor particles does not involve the mobilization of significant amounts of cellular phospholipid. Taken together, the results of these efflux studies suggest that ABCG1 generates a pool of cellular cholesterol at the cell surface that can transfer, without significant amounts of phospholipids, to extracellular acceptors with a lipid membrane surface.

### 3.4. Cycloheximide-Treated ABCG1-Expressing Cells Maintain Sufficient Plasma Membrane and Endosomal ABCG1 to Sustain ABCG1-Mediated Cellular Sterol Efflux

**Figure 6:**
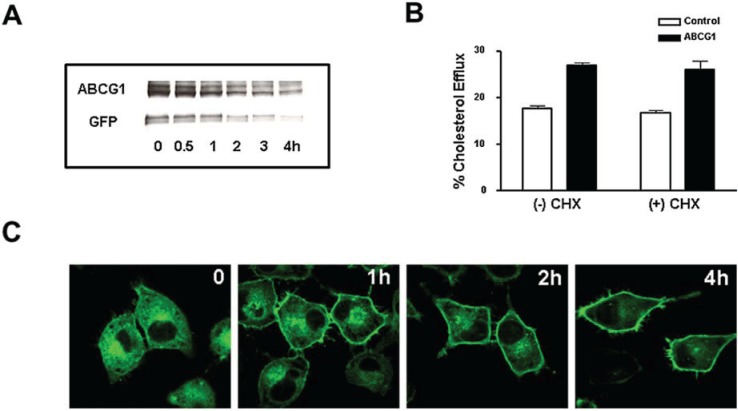
ABCG1-GFP at the cell surface and endosomes mediates cellular cholesterol efflux. (**A**) ABCG1 cells were incubated with 100 µg/mL cycloheximide (CHX) for the indicated times and extracted protein was immunoblotted with antibodies against *N*-terminus ABCG1 and GFP as described in “Experimental Section”. Note the marked decrease in total cellular ABCG1-GFP with time; (**B**) Control and ABCG1-GFP stably expressing HeLa cells were labeled with ^3^H-cholesterol and efflux to 50 µg/mL HDL in the absence or presence of CHX was assessed as described in “Experimental Section”. Note that CHX had little if any effect on ABCG1-mediated cellular cholesterol efflux. Two-way ANOVA analysis using multiple comparisons revealed that CHX did not induce any significant difference in efflux in either control or ABCG1 cells (*p* < 0.5 and *p* < 0.7, respectively), and that efflux in ABCG1 cells was significantly increased compared to control cells, with or without treatment (*p* < 0.0001, in all cases). All values represent mean ± S.D. Data shown is representative of at least three replicate experiments; (**C**) Compared to untreated ABCG1-GFP cells (0), treatment with CHX for 1, 2, and 4 h progressively decreases ABCG1-GFP (*green*) in the ER. Note that ABCG1 expression at the cell surface and endosomes is maintained up to 4 h after CHX treatment.

To assess the trafficking of ABCG1 from the ER to the cell surface and late endosomes, cells were treated with cycloheximide (CHX) to block protein synthesis. As anticipated, CHX reduced cellular levels of ABCG1-GFP (Figure 6A). Interestingly, cellular ABCG1-GFP was notably decreased after 1–2 h, and was greatly reduced by 4 h. Based on densitometric analysis of the ABCG1 Western blot shown in Figure 6A, ABCG1 had an apparent t_1/2_ of ~3.3 h. Despite the reduction in total cellular levels of ABCG1, ABCG1-mediated cellular cholesterol efflux was unaltered by CHX (Figure 6B). Confocal microscopy revealed that after 1–2 h, CHX markedly reduced the abundant ER pool of cellular ABCG1, and concomitantly increased the pool of ABCG1 on the cell surface and in endosomes (Figure 6C). Even up to 4 h, ABCG1 was retained on the cell surface, albeit at markedly reduced levels. Surprisingly, some cells retained markedly high levels of ABCG1 at the PM (Figure 6C). These findings suggest that concomitant with the CHX-induced block in ABCG1-GFP synthesis, the pre-existing pool of ABCG1-GFP in the ER trafficked to the plasma membrane, and then, to late endosomes. This pool of ER-derived ABCG1-GFP trafficked to the cell surface, where it sustained enhanced cholesterol efflux to HDL (Figure 6B). Taken together, these findings suggest that ABCG1 functions at the cell surface and endosomes to promote the efflux of cellular cholesterol to HDL.

### 3.5. Brefeldin A and U18666A Block both Delivery of ABCG1-GFP to the Cell Surface and ABCG1-Mediated Cellular Cholesterol Efflux

**Figure 7:**
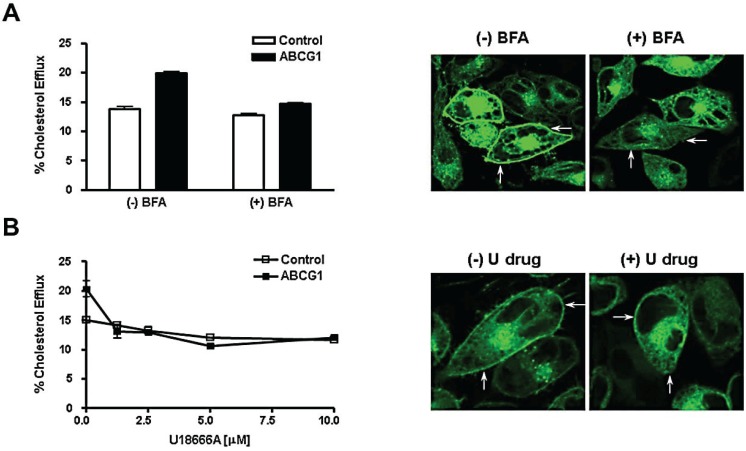
ABCG1-mediated cholesterol efflux requires delivery to the plasma membrane and endosomes. (**A**) Brefeldin A blocks ABCG1-mediated cellular cholesterol efflux. Control and ABCG1-GFP cells were labeled with ^3^H-cholesterol and efflux to 50 µg/mL HDL in the absence or presence of Brefeldin A (BFA) was assessed as described in “Experimental Section”. Two-way ANOVA analysis using multiple comparisons revealed that efflux from ABCG1 cells was significantly increased compared to control cells in the absence of BFA (*p* < 0.0001), that BFA did not induce any significant difference in efflux in control cells (*p* < 0.08) and that efflux from BFA-treated ABCG1 cells was significantly decreased compared to untreated ABCG1 cells (*p* < 0.0001). All values represent mean ± S.D. Data shown is representative of at least three replicate experiments. ABCG1-GFP stably expressing HeLa cells were incubated in the absence ((−) BFA) or presence ((+) BFA) of BFA for 4 h and then washed, fixed and observed by confocal microscopy as described in “Experimental Section”. Note that ABCG1-GFP is present in the ER in BFA-treated cells, but is markedly reduced at the cell surface (*arrows*) and in endosomes; (**B**) U18666A blocks ABCG1-mediated cellular cholesterol efflux. Control and ABCG1-GFP stably expressing HeLa cells were labeled with ^3^H-cholesterol, and efflux to 50 µg/mL HDL in the absence or presence of U18666A at the indicated concentrations was assessed as described in “Experimental Section”. Note that 1.25 µM U18666A blocks ABCG1-mediated cellular cholesterol efflux. Unpaired two-tail t-tests revealed that in the absence of U18666A, ABCG1 cellular cholesterol efflux was significantly greater than control cells (*p* < 0.003), and that with 1.25 µM U18666A treatment, ABCG1-mediated efflux was not significantly different than that of treated control cells (*p* < 0.2). All values represent mean ± S.D. Data shown is representative of at least three replicate experiments. ABCG1-GFP stably expressing HeLa cells were incubated in the absence ((−) U drug) or presence ((+) U drug) of 10 µM U18666A for 4 h and then washed, fixed, and observed by confocal microscopy as described in “Experimental Section”. Note that ABCG1-GFP is present in the ER in U18666A-treated cells, but is markedly reduced at the cell surface (*arrows*) and in endosomes.

We tested whether delivery of ABCG1-GFP to the cell surface is required for ABCG1-mediated cholesterol efflux using BFA to block transport along the secretory pathway. BFA causes the Golgi to fuse with the endoplasmic reticulum and blocks vesicular transport of newly synthesized protein to the cell surface [26]. Thus, BFA would be expected to cause newly-synthesized ABCG1-GFP to accumulate in the fused Golgi-ER and to reduce the amount of ABCG1-GFP at the cell surface and endosomes. BFA specifically blocked ABCG1-mediated cholesterol efflux, since cholesterol efflux to HDL from ABCG1 cells was markedly decreased in the absence of an effect on efflux from control cells (Figure 7A). Confocal microscopy revealed that ABCG1-GFP distributed in BFA-treated cells in a cytosolic reticular pattern and was markedly reduced on the cell surface and endosomes of ABCG1-GFP (Figure 7A). These studies reveal that ABCG1 trapped in the hybrid ER-Golgi organelle by the action of BFA cannot promote efflux of cellular sterol to HDL, and moreover, that delivery of the ABCG1 transporter to the cell surface and late endosomes is required for ABCG1-mediated cholesterol efflux. We further tested the role that ABCG1 trafficking to the cell surface plays in ABCG1-mediated sterol efflux using the hydrophobic amine U18666A. This drug perturbs intracellular cholesterol trafficking at several sites in a dose-dependent manner [27]. As shown in Figure 7B, 1.25 µM U18666A blocked ABCG1-mediated cellular cholesterol efflux in the absence of an effect on non-ABCG1-mediated cellular cholesterol efflux. U18666A, like BFA, markedly reduced ABCG1-GFP at the cell surface and in late endosomes (Figure 7B) confirming that delivery of ABCG1 to these sites is required for ABCG1-mediated cellular cholesterol efflux. U18666A-induced perturbations in intracellular cholesterol trafficking reduced the delivery of ABCG1 to the cell surface and late endosomes, raising the intriguing possibility that ABCG1 trafficking along the secretory pathway may be cholesterol-dependent.

### 3.6. ABCG1-GFP Trapped in Late Endocytic Compartments Can Still Mediate Enhanced Cellular Cholesterol Efflux

**Figure 8:**
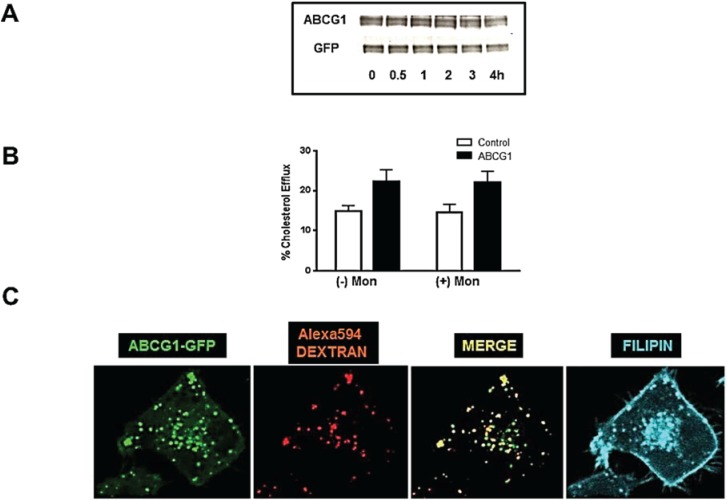
Endosomal ABCG1-GFP enhances cellular cholesterol efflux*.* PM-derived ABCG1 trapped in late endosomes/lysosomes by monensin enhances efflux to HDL. (**A**) ABCG1 cells were incubated with monensin for the indicated times and extracted protein was immunoblotted with antibodies against *N*-terminus ABCG1 and GFP as described in “Experimental Section”. Note total cellular ABCG1-GFP changes very little up to 4 h after treatment; (**B**) Control and ABCG1-GFP cells were labeled with ^3^H-cholesterol and efflux to 50 µg/mL HDL in the absence or presence of monensin was assessed, as described in “Experimental Section”. Note that monensin treatment had little if any effect on ABCG1-mediated cellular cholesterol efflux. Two-way ANOVA analysis using multiple comparisons revealed that monensin treatment itself did not induce any significant difference in efflux in either control or ABCG1 cells (*p* < 1.0 and *p* < 1.0, respectively), and that efflux in ABCG1 cells was significantly increased compared to control cells, with or without treatment (*p* < 0.0001, in all cases). All values represent mean ± S.D. Data shown is representative of at least three replicate experiments; (**C**) Confocal microscopy reveals that ABCG1-GFP (*green*) accumulates in intracellular vesicles, which partially colocalize with endocytosed Alexa594 dextran (*red*) used as a marker for late endocytic vesicles, seen as *yellow* vesicles in the merged image. The ABCG1-late endocytic vesicles are cholesterol-enriched, as revealed by filipin staining (*blue*).

Monensin [28], like brefeldin A, blocks delivery of newly-synthesized protein to the cell surface and thus would be expected to reduce surface expression of ABCG1-GFP, as well as possibly reduce HDL-mediated cholesterol efflux. In addition, monensin blocks protein degradation and trafficking out of late endosomes and lysosomes, and thus, is anticipated to cause PM-derived ABCG1-GFP to accumulate in late endosomes and lysosomes [28]. As shown in Figure 8A, monensin had little effect on total cellular ABCG1 protein levels. Surprisingly, monensin treatment of ABCG1 cells had little, if any, effect on HDL-mediated sterol efflux (Figure 8B). Monensin treatment markedly reduced ABCG1 at the cell surface, while markedly increasing the amount of ABCG1 in late endosomes/lysosomes marked with endocytosed Alexa594-dextran, as revealed by confocal microscopy (Figure 8C). Taken together, these results are consistent with a monensin-induced block in trafficking of newly-synthesized ABCG1-GFP from the Golgi to the cell surface, and accumulation of PM-derived ABCG1-GFP in late endocytic compartments. These findings suggest that ABCG1 trapped in late endocytic vesicles by monensin, unlike ABCG1 trapped in the ER by BFA, may play a role in mediating efflux of cellular cholesterol to extracellular HDL, and hence, the PM. Since monensin blocks vesicular trafficking out of late endosomes/lysosomes, it is possible that cellular cholesterol efflux in the presence of monensin represents movement of ABCG1-mobilized cholesterol from late endosomes/lysosomes to the PM by a non-vesicular-mediated pathway. Consistent with this notion, we previously reported that monensin traps PM-derived ABCA1 in late endosomes/lysosomes, and blocks ABCA1-mediated cellular cholesterol to apoA-I, by eliminating ABCA1 at the PM and by blocking retrograde vesicular trafficking required for the secretion of apoA-I potentially lipidated by ABCA1 in the lumen of late endosomes/lysosomes [1].

### 3.7. ABCG1-GFP Increases Endocytic Uptake of Fluid Phase Markers into Late Endocytic Compartments

**Figure 9:**
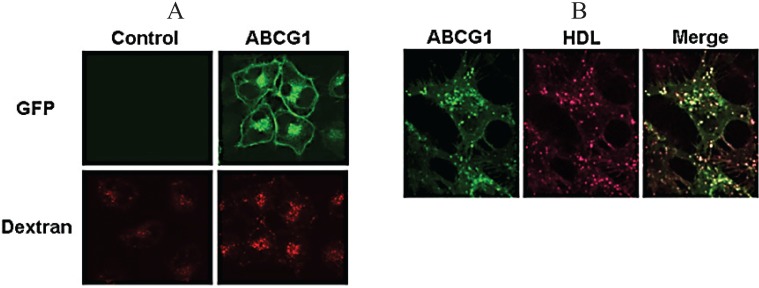

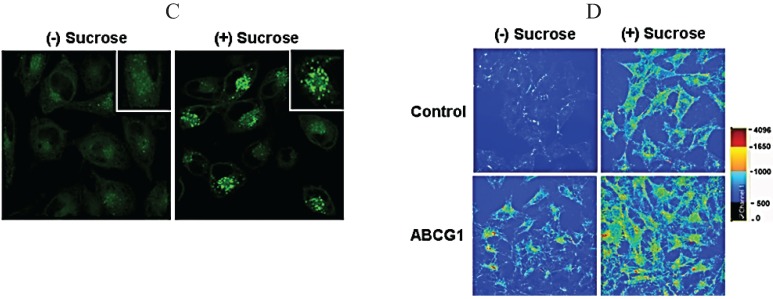
ABCG1 enhances endocytosis. (**A**) ABCG1 enhances endocytosis of fluorescent dextran. Control and ABCG1 cells were labeled with Alex594-dextran as described in “Experimental Section.” Note the marked increased uptake of fluorescent dextan (*red*) in ABCG1 cells; (**B**) Endocytosis of HDL in ABCG1-containing vesicles. ABCG1 cells were incubated with Alexa568-tagged HDL for 4 h as described in “Experimental Section.” Note that fluorescent HDL (*red*) colocalizes with ABCG1 late endosomes (*green*), seen as *yellow* vesicles in the merged image; (**C**) ABCG1 accumulates in sucrosomes. ABCG1 cells were incubated with 60 mM sucrose for 24 h, washed, fixed and imaged. Note the abundant accumulation of PM-derived ABCG1 in sucrosomes. *Insets*: Higher magnification of single ABCG1 cells; (**D**) ABCG1 increases endocytosis of HDL. Control and ABCG1 cells were incubated with Alexa568-tagged HDL with, or, without sucrose pre-treatment. Confocal images are color-coded according to fluorescence intensity (see scale on *right*). Note (i) the marked retention of endocytosed HDL late endosomes of ABCG1 cells compared to controls ((−) sucrose); (ii), increased HDL retention in sucrosomes in both control and ABCG1 cells ((+) sucrose), and; (iii), the notably increased retention of HDL in ABCG1 sucrosomes compared to controls.

As noted earlier, endocytosed fluorescent dextran accumulates in ABCG1-containing late endocytic compartments (Figure 2b). As shown in Figure 9, ABCG1 cells exhibited a marked increase in uptake of both fluorescent dextran (Figure 9A) and Alexa 568-tagged HDL (Figure 9D) into ABCG1-containing perinuclear late endocytic vesicles (Figure 9B). We next tested the effect of sucrosome formation on HDL endocytosis. Sucrosomes are osmotically swollen late endosomes/lysosomes, formed after prolonged endocytic uptake of sucrose, that are unable to traffic PM-derived cargo to other destinations [29,30]. As shown in Figure 9C, sucrose treatment led to the formation of sucrosomes containing markedly increased levels of ABCG1. As shown in Figure 9D, entrapment of endocytosed fluorescent HDL in lysosomes in the presence of sucrose revealed a dramatic increase in accumulation of HDL in ABCG1 lysosomes compared to both non-sucrose-treated ABCG1 cells, as well as sucrose-treated control cells. This increased uptake in ABCG1 cells likely represents an increased rate of endocytic vesicle formation that may be a function of increased cholesterol content at the PM of ABCG1 cells. However, based on our studies, we cannot exclude the possibility that the increased retention of endocytosed fluid-phase markers in ABCG1-late endosome/lysosomes may in part reflect decreased clearance from late endosomes/lysosomes. The observed trafficking of endocytosed HDL in ABCG1 late endocytic vesicles raises the intriguing possibility that ABCG1 may promote intravesicular transfer of endosomal cholesterol to HDL, which may then efflux cholesterol from the cell upon HDL resecretion.

### 3.8. ABCG1-Mediated Cellular Cholesterol Efflux to HDL is Maintained When Cellular Sphingomyelin is Depleted

**Figure 10:**
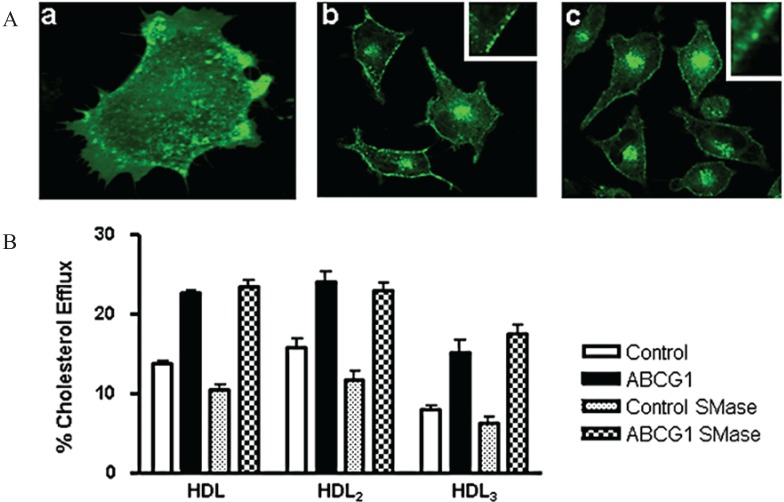
Sphingomyelin hydrolysis increases cycling of ABCG1 between the cell surface and endosomes and ABCG1-mediated cholesterol efflux to HDL. (**A**) Endovesiculation of cell surface ABCG1-GFP. Confocal micrographs of ABCG1-GFP cells treated with sphingomyelinase (SMase) for 10 min (**a**,**b**) or 30 min (**c**). Insets in (**b**) and (**c**) show numerous subsurface endovesicles containing ABCG1-GFP. Single frame from Movie 5 (**a**) reveals massive endovesiculation of cell surface ABCG1-GFP; (**B**) Control and ABCG1-GFP stably expressing HeLa cells were labeled with ^3^H-cholesterol and efflux to 50 µg/mL HDL, HDL_2_, or HDL_3_ in the absence or presence of 0.1 U/mL SMase was assessed as described in “Experimental Section”. Note that SMase decreases cholesterol efflux in control cells but does not alter net cellular cholesterol efflux in ABCG1-GFP cells. One-way ANOVA analyses using multiple comparisons revealed that SMase significantly reduced efflux from control cells to HDL, HDL_2_, and HDL_3_ (*p* < 0.0001, *p* < 0.0001, and *p* < 0.05, respectively), and that cholesterol efflux to HDL, HDL_2_, and HDL_3_ from SMase-treated ABCG1 cells was significantly increased compared to SMase-treated control cells (*p* < 0.0001, in all cases). SMase treatment did not significantly increase cholesterol efflux to HDL or HDL_2_ (*p* < 0.06 and *p* < 0.4), but did increase efflux to HDL_3_ (*p* < 0.003). All values represent mean ± S.D. Data shown is representative of at least three replicate experiments.

To further probe the cell surface distribution of ABCG1, sphingomyelinase (SMase) digestion of cell surface sphingomyelin (SM) was used to induce rapid endovesiculation [31] of ABCG1 at the plasma membrane. Within 10 min of SMase treatment, plasma membrane-derived ABCG1-GFP was observed to redistribute to subsurface endovesicles (Figure 10A). Time-lapse confocal microscopy dramatically illustrates the rapid generation of these PM-derived ABCG1-GFP-containing endovesicles, and the rapid recycling of these vesicles back to the cell surface (Figure 10A & Movie 5). SMase treatment reduced HDL-mediated cellular cholesterol efflux from control cells, but not from ABCG1 expressing cells (Figure 10B). Since the non-ABCG1-mediated efflux pathway would also be expected to decrease in the ABCG1 cells, SMase apparently diverted the SM-associated pool of cholesterol available for efflux to the ABCG1-mediated cellular cholesterol efflux pathway. These studies establish that SM at the cell surface is not required for ABCG1-mediated cellular cholesterol efflux. Interestingly, when the rate of ABCG1 cycling between the cell surface and endosomes was markedly increased by SMase, the transporter maintained its ability to promote cholesterol efflux, suggesting that prolonged residence on the cell surface is not required for ABCG1-mediated sterol efflux, and/or that endosomal ABCG1 functions in cellular cholesterol efflux.

## 4. Conclusions

We have studied the cellular localization, trafficking and site(s) of function of the human ABCG1 transporter in a heterologous expression system consisting of a control parental HeLa cell line which lacks endogenous expression of ABCG1, and a HeLa cell line that stably expresses GFP-tagged ABCG1. The enhancement of cellular cholesterol efflux to HDL, HDL_2_, HDL_3_, or LDL by GFP-tagged ABCG1 was similar to that reported for non-tagged ABCG1 [12,13,14,32], thus establishing that fusion of GFP to the C-terminus of the transporter does not alter its functionality. ABCG1 expression increased both the size of the cholesterol pool available for efflux and the rate constant for efflux, as previously reported [25]. ABCG1-GFP did not enhance efflux of either cellular cholesterol or phospholipid to apoA-I, confirming that ABCG1 and ABCA1 mediate fundamentally different alterations in membrane lipid organization. ABCG1-GFP enhanced cellular cholesterol efflux to PC liposomes, demonstrating that a lipid surface is the minimal structural requirement for an acceptor of ABCG1-mobilized cellular cholesterol.

We have shown that ABCG1-GFP traffics along the secretory pathway from the ER to the cell surface and then along the endocytic pathway to late endosomes/lysosomes. Pharmacologically-induced blocks in transport to the cell surface were observed to block the ABCG1-mediated increase in cellular cholesterol efflux, establishing that delivery to the cell surface is required for ABCG1 to mediate cellular cholesterol efflux. We also observed that the hydrophobic amine U18666A, which perturbs intracellular cholesterol trafficking, blocked ABCG1 trafficking along the secretory pathway, raising the intriguing possibility that ABCG1 secretion may be cholesterol-dependent.

ABCG1-GFP trapped in late endosomes/lysosomes by monensin still enhanced cellular cholesterol efflux, consistent with a role for endosomal ABCG1 in cellular cholesterol efflux. Our findings are similar to recent studies that also demonstrated that ABCG1 functions in late endosomal compartments to modulate intracellular cholesterol trafficking [17]. Our studies have provided additional evidence that ABCG1 in late endosomes can mobilize cholesterol for non-vesicular-mediated trafficking pathways to the cell surface and, strongly suggest that ABCG1 mobilizes cholesterol on both leaflets of cellular membrane bilayers for transfer to an acceptor. 

3-D and 3-D time-lapse confocal microscopy revealed an extensive network of ABCG1-containing perinuclear and subsurface endosomes that cycle between these sites and which make frequent contact with each other as well as the cell surface. Given that a lipid surface can serve as an extracellular acceptor of ABCG1-mobilized cholesterol, it is tempting to speculate that transfer of ABCG1-mobilized membrane cholesterol may occur upon collision of endosomes with one another or with the cell surface. Thus, ABCG1 appears to increase the availability of membrane cholesterol to traffic by membrane interactions [33], or possibly by non-vesicular cytosolic cholesterol carrier proteins, such as the START and OSBP proteins [34]. Indeed, recently ORP1L was implicated in potentially modulating ABCG1-mobilized pools of cellular cholesterol [35]. Since removal of membrane cholesterol may require binding of the carrier protein to the membrane, removal of ABCG1-mobilized membrane cholesterol either by membrane collision or by cytosolic carrier proteins may involve similar mechanisms. However, it has been shown that increased ABCG1-mediated cholesterol efflux to HDL does not involve increased HDL binding to the cell surface [12] and that the transfer of cholesterol from cellular membranes to an acceptor involves desorption from the donor membrane, followed by aqueous diffusion and absorption by the acceptor [7]. One possibility is that ABCG1 may enhance availability of membrane cholesterol to acceptors by increasing the chemical potential of cholesterol, *i.e.*, the protrusion of cholesterol out of the membrane surface. The transfer of ABCG1-activated cholesterol to an acceptor may then involve either collisional or diffusional mechanisms.

We have confirmed the findings by Sankaranarayanan *et al.* [25] that ABCG1 expression increases both the size of the cholesterol pool available for efflux and the rate constant for efflux. The structural basis for these kinetic effects is now explained by our finding that ABCG1 expression increases the amount of cholesterol on the cell surface (increased cholesterol efflux pool). The increased efflux rate constant (enhanced cholesterol chemical activity and ability to desorb from the membrane) reflects the ABCG1-induced reorganization of the plasma membrane, as demonstrated in this and our accompanying paper [36].

In conclusion, our studies establish that ABCG1 residing on the cell surface and late endosomes mobilizes cholesterol at these sites for transfer to extracellular acceptors with a lipid surface and have provided several new insights into the mechanisms involved in ABCG1-mediated alterations in intracellular cholesterol trafficking.

## Supplementary Files

Supplementary File 1

Supplementary File 2

Supplementary File 3

Supplementary File 4

Supplementary File 5

Supplementary File 6
