# Supplementary material for: Cellular Localization and Trafficking of the Human ABCG1 Transporter
Source: Biology (Basel). 2014 Nov 14;3(4):781–800. doi: 10.3390/biology3040781 (PMC4280511; doi:10.3390/biology3040781)
Supplement: Supplementary File 1 [file biology-03-00781-s001.pdf]

Supplemental Data

Movie 1. ABCG1 Resides on the Cell Surface.

Maximum projection of confocal microscopic z-stack of GFP fluorescence through the entire volume of a single ABCG1-expressing cell. Data set was rendered to highlight cell surface fluorescence using Imaris software.

Movie 2. ABCG1 Resides on the Plasma Membrane and Late Endosomes.

Merged maximum projection image showing colocalization of fluorescent dextran in ABCG1-late endosomes (*yellow*). Note the abundant perinuclear and peripherally located ABCG1-late endosomes. The same data set in Movie 1 was rendered to reveal both cell surface and intracellular ABCG1-GFP fluorescence. ABCG1-GFP cells were incubated with Alexa594-dextran to label late endosomes as described in "Experimental Section", and imaged by confocal microscopy for GFP fluorescence (*green*) and dextran fluorescence (*red*).

Movie 3. ABCG1-Late Endosomes Shuttle between Perinuclear ABCG1-Late Endosomes and the Cell Surface.

Time-lapse confocal fluorescence microscopy of living ABCG1-GFP cells reveals plasma membrane and late endosomal localization of the transporter. Note that ABCG1-late endosomes in the perinuclear cluster interact with each other and the peripheral ABCG1-late endosomes interact with one another. In addition, peripheral ABCG1-late endosomes shuttle between the perinuclear late endosomes and, as denoted by the *arrowheads,* frequently make contact with the cell surface.

Movie 4. 3D-Time Lapse Confocal Fluorescence Imaging of Living ABCG1-Cells.

3D image stacks of GFP fluorescence of ABCG1-GFP cells were continuously imaged by confocal microscopy to reveal cell surface and late endosomal ABCG1. ABCG1-late endosomes are pseudo-colored as *yellow* spheres. Note the numerous peripheral ABCG1-late endosomes near the cell surface that make frequent contact with the plasma membrane. View this movie using the VLC media player.

Movie 5. Sphigomyelinase-Induced Endovesiculation of Plasma Membrane ABCG1.

Time-lapse fluorescence confocal microscopy of ABCG1 10 min after incubation with 0.1 U/mL sphingomyelinase. Note the rapid and massive formation of ABCG1-endovesicles from plasma membrane ABCG1-GFP and rapid trafficking and fusion of ABCG1-endosomes with the cell surface. ABCG1-endovesicles are seen to rapidly undergo fusion and fission with one another. Tubular ABCG1-endovesicles appear to traffic along cytoskeletal elements (see center of cell). The enlarged region in *white* highlights details of ABCG1-endovescular interactions and trafficking.
